# “Environmental risk factors associated with juvenile idiopathic arthritis associated uveitis: a systematic review of the literature”

**DOI:** 10.1186/s12348-021-00247-1

**Published:** 2021-05-24

**Authors:** Sarah L. N. Clarke, Katie S. Mageean, Henry Carlton, Gabriele Simonini, Gemma C. Sharp, Caroline L. Relton, Athimalaipet V. Ramanan

**Affiliations:** 1 MRC Integrative Epidemiology Unit, University of Bristol, Bristol, UK; 2 Population Health Sciences, Bristol Medical School, University of Bristol, Bristol, UK; 3 Department of Paediatric Rheumatology, Bristol Royal Hospital for Children, Bristol, UK; 4 Rheumatology Unit, Meyer Children Hospital, NEUROFARBA Department, University of Florence, Florence, Italy; 5 Translational Health Sciences, Bristol Medical School, University of Bristol, Bristol, UK

**Keywords:** Juvenile idiopathic arthritis, Uveitis, Risk factors, Environment, Vitamin D, Season, Allergy

## Abstract

**Background:**

Juvenile idiopathic arthritis associated uveitis (JIA-U) is the most common extra-articular manifestation of juvenile idiopathic arthritis (JIA) and carries considerable risk to vision. The aim of this systematic review was to synthesise evidence of environmental risk factors for JIA-U and identify risk factors which may be modifiable or used to stratify JIA patients.

**Methods:**

This systematic review was carried out in accordance with PRISMA guidelines. Four online databases - Cumulative Index of Nursing and Allied Health Literature, Web of Science, MEDLINE and Embase - were searched from database inception to 12th August 2020. Identified studies were screened by two independent reviewers against pre-defined inclusion and exclusion criteria. Data was extracted from all primary studies meeting inclusion criteria and independently checked.

**Results:**

We identified three studies from 895 unique records which met the inclusion criteria, each examining a different environmental risk factor. This systematic review includes 973, predominantly female, participants with JIA across these three studies. The use of allergy medication or documentation of “allergy”/“allergic” in the medical records was associated with an increased risk of JIA-U in all models presented. Vitamin D sufficiency was associated with reduced risk of JIA-U. There was insufficient evidence to support an association between seasonality and JIA-U.

**Conclusions:**

This review identifies a potential role for allergy and vitamin D in JIA-U. It also illustrates the paucity of data regarding environmental risk factors for JIA-U and highlights the need for further research to both identify additional risk factors and replicate existing findings.

## Background

Juvenile idiopathic arthritis (JIA) is the most common rheumatic condition of childhood and juvenile idiopathic arthritis associated uveitis (JIA-U) is the most common associated extra-articular manifestation. The estimated prevalence of JIA-U in JIA patients is 11–38% [1, 2]. However, prevalence varies by JIA subtype with estimates as high as 47% in oligoarticular JIA [3]. JIA-U is often asymptomatic but carries considerable risk to vision thus children with JIA undergo regular ophthalmic screening [4]. Guidelines for frequency and duration of JIA-U screening are based on existing clinical risk factors of gender, JIA subtype, age of JIA onset, and anti-nuclear antibody (ANA) and HLA-B27 status. However these lack specificity and children may still develop uveitis between screening visits [5].

JIA-U is considered to be a heterogenous disease, driven by both genetic and environmental factors. There has been increasing literature regarding genetic influences on JIA-U susceptibility [6] and the first genome wide association study of JIA-U has recently been published [7]. It has previously be noted that evidence of external (environmental) triggers for JIA-U is limited and environmental risk factors for all cause uveitis have been summarised [8]. However, the identification of specific risk factors for JIA-U will assist in improving the JIA-U screening programmes as well as potentially redefining treatment strategies for patients based on their individual risk profile. Thus, the aim of this systematic review is to identify environmental risk factors associated with JIA-U incidence which may either be modifiable or assist in risk stratification of JIA patients. To our knowledge this is the first systematic review examining environmental risk factors for JIA-U.

## Methods

This study follows guidance from the Preferred Reporting Items for Systematic Reviews and Meta-analyses (PRISMA) statement [9]. The protocol for this systematic review was pre-registered on PROSPERO (ID: CRD42017078306) [10] and can be accessed at https://www.crd.york.ac.uk/prospero/display_record.php? RecordID = 78,306.

### Literature searching

Literature searching of online bibliographic databases were carried out in order to identify literature on environmental risk factors associated with JIA-U. Expert advice from a database searching specialist was sought prior to designing the search strategy. Each search was carried out in four databases; MEDLINE and Embase via Ovid, Web of Science (WOS) via Clarivate Analytics and Cumulative Index of Nursing and Allied Health Literature (CINAHL) via EBSCOhost. Searches included literature from database inception until 12th August 2020. Table 1 shows the search strategy that was used for MEDLINE; the syntax was amended for use in other databases. All searches were limited to studies in English only.

**Table 1:** Search strategy for MEDLINE by OVID (syntax amended for use in other databases)

1	(juvenile adj2 arthritis).tw
2	Arthritis, Juvenile/
3	Uveitis/
4	uveitis.tw
5	iridocyclitis.tw
6	inflammat* ocul*.tw
7	inflammat* eye.tw
8	non-infectious uveitis.tw
9	autoimmune uveitis.tw
10	Risk Factors/
11	Environment/
12	Seasons/
13	Postpartum Period/
14	Pregnancy/
15	Birth order/
16	age factors/ or maternal age/
17	paternal age/
18	Socioeconomic Factors/
19	Demography/
20	Infection/
21	Communicable Diseases/
22	Bacterial Infections/
23	risk.tw
24	environmen*.tw
25	perinatal.tw
26	(season* adj3 birth).tw
27	smok*.tw
28	virus.tw
29	1 or 2
30	3 or 4 or 5 or 6 or 7 or 8 or 9
31	10 or 11 or 12 or 13 or 14 or 15 or 16 or 17 or 18 or 19 or 20 or 21 or 22 or 23 or 24 or 25 or 26 or 27 or 28
32	29 and 30 and 31

### Study selection

References (including abstracts where available) were downloaded into Endnote X9 (Clarivate Analytics) and duplicates were removed. All unique references were uploaded to Rayyan [11] and underwent title and abstracts screening by two independent reviewers (SC and KM/HC) against the inclusion and exclusion criteria listed in Table 2. The full texts of potentially relevant studies identified during title and abstract screening were retrieved. These articles were again independently screened by two reviewers (SC and KM/HC). Bibliographies of review articles were also hand-searched to identify other potentially relevant studies and expert advice was sought from AR regarding key studies which may not have been captured within our searches. Any discrepancies during screening were resolved by discussion and/or involvement of the third reviewer (AR). Where a study was felt to be highly relevant but key inclusion/exclusion criteria were not reported, study authors were contacted prior to a final decision being made.

**Table 2:** Inclusion and exclusion criteria for studies

Domain	Inclusion criteria	Exclusion criteria
Study language	English	Not English
Study type	Systematic review Observation study (cohort, case-control, cross-sectional)	Non-systematic review article Clinical trial Animal study In vitro study Ex vivo study Case report
Study population	Patients with juvenile idiopathic arthritis associated uveitis (JIA-U), diagnosed using any recognised uveitis diagnostic criteria [e.g. International Uveitis Study Group (IUSG), Standardised Uveitis Nomenclature (SUN), International Classification of Diseases (ICD)] who also meet diagnostic criteria for juvenile idiopathic arthritis (JIA), diagnosed using any recognised criteria [e.g. American College of Rheumatology (ACR), EULAR,, International League of Associations for Rheumatology (ILAR), ICD] PLUS Onset of disease at or before 16 years of age	Adults (defined as age > 16 yrs)
Study comparator	General population without JIA or JIA-U	Other rheumatic, autoimmune or inflammatory disease
Study risk factor	Environmental risk factors (including patient, familial and perinatal)	Non-environmental risk factors (e.g. genetic, ethnic/racial, familial aggregation)

### Data extraction

A data extraction form was created and piloted in Microsoft Excel by SC and KM. Data was extracted from each eligible study by SC and independently checked by KM/HC. Data extraction consisted of:

- First author
- Study title
- Year of publication
- Study country
- Participant demographic characteristics
- Sample size
- Diagnostic criteria used
- Length of follow-up
- Outcomes
- Event rates for unexposed/exposed cases and controls
- Risk factors listed in univariate and multivariate analyses
- Covariates adjusted for in analysis
- Source of risk factor ascertainment
- JIA and JIA-U subtypes included
- Newcastle-Ottawa scale scores

For consistency between studies, the extracted sample size was the sample size of the largest analysis undertaken within a study. All data is presented as odds ratio and 95% confidence intervals where possible.

### Risk of bias (quality) assessment

The methodological quality and risk of bias of included studies was assessed using the Newcastle-Ottawa Scale (NOS) for case-control and cohort studies [12]. Studies were independently scored by SC and KM/HC and any discrepancies were resolved by discussion and/or involvement of a third reviewer (AR). A risk of biases table was created summarising the star rating of included studies (maximum score of 9 stars reflecting the highest quality).

### Data synthesis

None of the included studies examined overlapping risk factors, thus there was no data suitable for the quantitative meta-analysis. Therefore, as specified in our protocol, the data within this systematic review is synthesised in narrative form. The principal summary measure for this systematic review is unadjusted and adjusted odds ratio. Where this was not reported by a study, or was not possible to derive from the raw data presented within a study, we used the study reported summary measure for data synthesis.

## Results

### Study selection

**Fig. 1:**
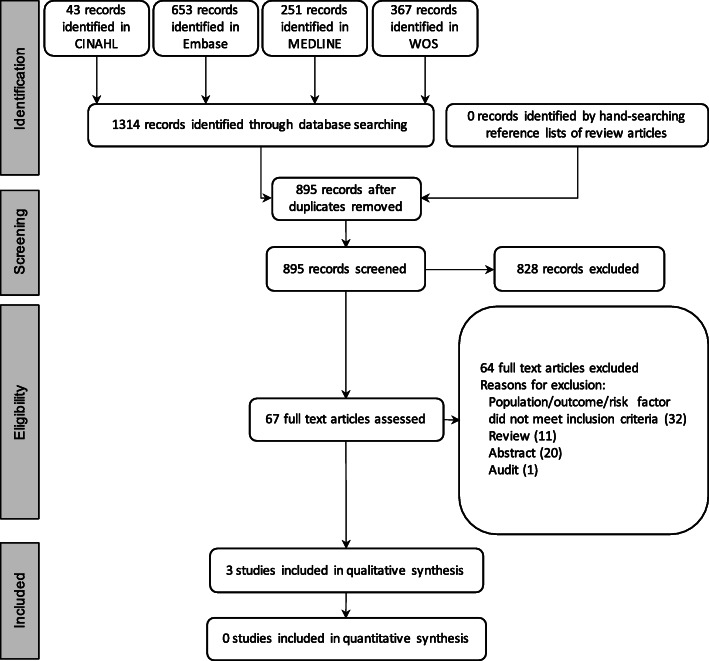
PRISMA flow diagram of study selection

Searches of CINAHL, Embase, MEDLINE and WOS on 12th August 2020 identified 1314 JIA-U studies. After collation, 419 studies were identified as duplicates by Endnote X9. The majority of studies were excluded during title and abstract screening. In total, 67 studies were assessed in full and of these, three studies met the inclusion criteria listed in Table 2. The flowchart for selection of included studies and specific reasons for exclusion is shown in Fig. 1.

### Characteristics of included studies

The characteristics of the three included studies are shown in Table 3. All studies were retrospective cohort studies. Due to publication date and country of origin, the studies are presumed to encompass non-overlapping samples thus this systematic review includes a total sample size of 973 participants. All studies reported female predominance to JIA-U within the JIA population. Cole et al [14] and Sengler et al [15] reported statistically significant differences in the mean age of JIA onset between JIA-U cases and controls; patients who developed JIA-U had younger age of JIA onset. Zulian et al [13] also reported a lower mean age of JIA onset in patient who developed mild JIA-U however this did not reach statistical significance. All three studies utilised different JIA and JIA-U diagnostic criteria and included different JIA subtypes.

**Table 3:** **Characteristics of included studies.** *Demographic data for this study was stratified by uveitis severity. ICD, International Classification of Diseases, ILAR, International League of Associations for Rheumatology; IUSG, International Uveitis Study Group; JIA, juvenile idiopathic arthritis, JIA-U, juvenile idiopathic arthritis associated uveitis; SD, standard deviation; SUN, Standardisation of Uveitis Nomenclature

Study	Study type	Country	Sample size	Age of JIA onset, cases (mean ± SD, years)	Age of JIA onset, controls (mean ± SD, years)	Sex of cases (Female, %)	Sex of controls (Female, %)	Risk factors	Diagnostic criteria	JIA subtypes included	JIA-U subtypes included
*Zulian, 2002* [13]	Cohort	Italy	316	2.42 ± 1.5 (mild)*, 3.17 ± 1.75 (severe)*	3.08 ± 1.75	89.01 (mild)*, 74.36 (severe)*	78.26	Seasonality	ILAR-1997, IUSG 1987	Oligoarticular JIA only	No/mild/severe uveitis
*Cole, 2013* [14]	Cohort	USA	297	7.2	10.2	76.19	65.10	“Allergy” or “Allergic” in medical records, allergy medication use	ICD-9	All (ICD-9 codes 696.0, 714.0, 714.2, 714.3, 714.9, 720.2, 720.9)	Acute and chronic iridocyclitis (ICD-9 codes 364.00 and 364.10)
*Sengler, 2018* [15]	Cohort	Germany	360	4.0 ± 2.9	7.8 ± 4.6	73.7	66.2	Vitamin D	ILAR 2001, SUN 2005	All	Any

### Quality assessment and risk of bias

The NOS score of the included studies ranged from five to seven, with the full breakdown of NOS scores shown in Table 4. All studies lost points for the length of participant follow-up.

**Table 4:** Newcastle Ottawa scale scores for included studies

Study ID	Selection						Comparability		Outcome					Total
	Representativeness of exposed cohort		Non-exposed cohort from same community as exposed cohort	Ascertainment of exposure		Outcome of interest not present at start of study	Study controls for socioeconomic status	Study controls for maternal age/year of birth	Assessment of outcome		Follow up long enough for outcome to occur	Adequacy of follow up		
	Truly	Somewhat		Secure record	Blinded interview				Independent blind assessment	Record linkage		Complete follow up	> 95% follow up or description provided of loss to follow-up	
Zulian, 2002 [13]	1	0	1	1	0	1	0	1	0	1	0	1	0	7
Cole, 2013 [14]	1	0	1	1	0	0	0	1	0	1	0	0	0	5
Sengler, 2018 [15]	1	0	1	1	0	0	1	1	0	1	0	0	0	6

### Environmental risk factors

Three environmental risk factors from three studies were examined – seasonality, allergy and vitamin D, see Table 5. Of these, only seasonality was reported in an unadjusted model; unadjusted estimates were not reported for allergy and vitamin D, nor was sufficient raw data provided in the studies to derive this estimate. Zulian et al [13] reported winter to be associated with an increased risk of JIA-U, autumn and spring to be associated with a decreased risk of JIA-U and summer to show no association with JIA-U. The confidence interval around these estimates were not reported, nor was sufficient event rate data available to derive these. In all cases, the *p* value was > 0.05 (exact values not reported), providing insufficient evidence to support an association between seasonality and JIA.

**Table 5:** **Risk factors associated with JIA-U.** ^a^reported point estimate, ^b^confidence intervals not reported, p-value > 0.05, ^c^adjusted for race and gender, ^d^adjusted for race, gender, age, oligo subtype, ANA, RF, psoriasis, ^e^reported as HR, ^f^adjusted for MTX and uveitis risk factors (age of JIA onset, female sex, oligoarticular JIA and ANA positivity), ^g^when analysed by patients who were sampled prior to uveitis diagnosis the results were HR 0.95, 95% CI 0.92–1.00. ANA, anti-nuclear antibody; CI, Confidence interval; HR, hazard ratio; MTX, methotrexate; NR, not reported; OR, odds ratio; RF, rheumatoid factor; 25(OH) D, 25-hydroxyvitamin D

Study ID	Risk factor	Definition	Univariate OR (95% CI)	Bivariate OR (95% CI)	Multivariable OR (95% CI)
Zulian, 2002 [13]	Seasonality	Autumn	0.85^a,b^	NR	NR
		Summer	1.00^a,b^	NR	NR
		Winter	1.39^a,b^	NR	NR
		Spring	0.96^a,b^	NR	NR
Cole, 2013 [14]	Allergy	“Allergy” in clinical notes	NR	2.14 (1.08–4.27)^c^	NR
		“Allergic” in clinical notes	NR	2.68 (1.34–5.55)^c^	NR
		Any allergy medication	NR	2.92 (1.47–5.91)^c^	2.54 (1.22–5.4)^d^
Sengler, 2018 [15]	Vitamin D	1 ng/mL increase in 25(OH) D level > 22.1 ng/mL	NR	NR	0.95 (0.91–0.99)^e,f,g^

Both allergy and vitamin D were examined in an adjusted model. Cole et al [14] reported the association between allergy and JIA-U by examining the documentation of “allergy” or “allergic” within the clinical records, and the documented use of allergy medication. Bivariate analysis demonstrated that “allergy” or “allergic” in the medical records, and the use of allergy medications were all associated with an increased risk of JIA-U (Table 5). The use of allergy medication was also reported to be associated with increased risk of JIA-U (OR 2.54, 95% CI 1.22–5.40) in a model adjusted for race, gender, age, oligoarticular subtype, ANA, rheumatoid factor (RF) and psoriasis. Sengler et al [15] investigated the risk of JIA-U according to vitamin D status. They reported the risk of JIA-U to be inversely correlated with mean vitamin D level in a multivariable model (HR 0.95, 95% CI 0.91–0.99) adjusted for methotrexate use and existing uveitis risk factors (including at JIA onset, female sex, oligoarticular JIA, and ANA positivity). This association persisted when the study population was restricted to those participants for whom vitamin D status was measured prior to JIA-U onset.

## Discussion

The literature search strategy was designed to capture environmental risk factors for JIA-U in the broadest sense and identify all relevant literature regarding JIA-U risk. Nevertheless, the identification of only three studies highlights the unmet research need in this area.

The demographic characteristics of the included studies illustrate that they are representative of the JIA community; JIA-U is reported to be more common in younger, female JIA patients as is seen here. The NOS scores of the studies suggest the studies are of generally of good quality. However, all studies lost points for failing to meet adequate follow-up; length of follow up was reported to be 3 years by Sengler et al and a minimum of 2 years by Zulian et al*,* but was not reported in the study by Cole et al. The natural history of JIA-U suggests that 90% of JIA patients develop JIA-U within the first 4 years after JIA diagnosis [16, 17]. Thus, risk factor studies in JIA-U should ideally follow up participants for at least this period of time. These findings are likely to reflect the challenges of performing robust studies in rare diseases where patient cohorts are small and where appropriate datasets to identify and analyse risk factors robustly may be lacking or limited.

JIA-U is generally considered to be a complex disease, influenced by both genetic and environmental risk factors. The use of broad literature search terms used in this systematic review captured 692 de-duplicated records yet identified only three studies which met the inclusion criteria. Correspondingly we only identified three environmental risk factors which had been examined with regards to JIA-U incidence, none of which overlapped sufficiently to enable statistical comparison. Whilst the primary limitation to performing meta-analysis was lack of replicated risk factors, it should be noted that the included studies used different criteria to ascertain JIA cohorts and define JIA-U status. A number of different criteria have been defined and amended to diagnose JIA over the past 50 years [18] alongside ongoing revisions of the ICD codes. Furthermore, uveitis criteria have also evolved over this time [19]. These factors make the synthesis of historic data challenging and should be considered in planning future studies.

There is increasing recognition of the seasonal variation in both the incidence and disease activity of a number of autoimmune diseases. However, the influence of seasonality on autoimmunity is likely complex and multifactorial. Seasonal variation is observed with a number of factors such as infections, ultraviolet light exposure, vitamin D and melatonin [20]. Winter is associated with viral respiratory pathogens, spring is associated with bacterial respiratory pathogens such as *Mycoplasma pneumoniae* [21] and late summer/early autumn is associated with increased prevalence of viral pathogens such as Enterovirus [22]. Seasonal viral infections are postulated to contribute to autoimmunity via mechanisms such as molecular mimicry, epitope spreading and bystander activation [23]. In addition, seasonal sunlight exposure markedly influences vitamin D levels [24], which peak in the summer/autumn and reach a nadir in the winter. Vitamin D has a number of effects on immune function [25] though evidence for hypovitaminosis D as a risk factor for autoimmune disease incidence, with the exception of multiple sclerosis, is limited. Nevertheless the finding of an association between vitamin D and JIA-U is also supported by a study examining the role of vitamin D in non-infectious uveitis in adults (sample size 3348) which found a 21% reduction in uveitis risk with normal versus low vitamin D levels (≤20 ng/mL) [26]. Whilst this uveitis cohort does not specifically examine JIA-U, it includes participants with other T-cell driven uveitides. Furthermore, the concordant directions of effect seen in the association between seasonality and JIA-U add strength to the association with vitamin D; Zulian et al [13] found autumn to be associated with decreased risk of JIA-U and winter to be associated with an increased risk of JIA-U (albeit in univariate analysis with a *p*-value > 0.05) and Sengler et al [15] reported vitamin D sufficiency to be significantly associated with decreased risk of JIA-U. There are a number of possible reasons for these findings; hypovitaminosis D may itself be a risk factor for JIA-U, it may be a marker of another seasonal exposure, or several seasonal factors may act in combination (e.g. hypovitaminosis D and an infectious agent). Additionally, these associations may be spurious findings given the limited data seen here. Further studies to corroborate and delineate these associations, and allow meta-analysis of the results are warranted. However, such studies are challenging; the asymptomatic nature and insidious onset of JIA-U means the vitamin D level or season at diagnosis may not reflect the vitamin D level or season at JIA-U onset. This temporality needs consideration when designing future studies, which should ideally take place in the context of regular and frequent ophthalmic screening.

Traditionally allergy and autoimmunity were felt to represent different facets of a perturbed immune system under the Th1/Th2 paradigm – with autoimmune diseases presenting in those with a more T helper cell type 1 (Th1) predisposition and allergies presenting in those with a more T helper cell type 2 (Th2) predisposition [27, 28]. However, this paradigm has been challenged in more recent years with the discovery of Th17 cells [29]. Recent data has been shown that rather than being protective, patients with allergic and atopic conditions are at increased risk of developing autoimmunity [30]. We found limited data on the association between allergy and JIA-U, however the direction and magnitude of this association is comparable with estimates of other allergy/atopy/autoimmune associations reported by Krishna et al [30]. Given the immunopathogenic basis of allergic/atopic and autoimmune diseases, it is difficult to ascertain whether associations between these two disease types are evidence of a causal relationship (and in which direction) or whether this association is a correlation due to, for example, underlying perturbation of immune function. Nevertheless, given the increasingly recognised association between autoimmune and allergic traits, further studies and resultant meta-analyses in JIA-U may provide support for clinicians to highlight JIA patients with increased JIA-U risk based on their allergy status.

The reasons for the high incidence of uveitis in patients with JIA are poorly understood. Whilst identification and examination of the environmental risk factors overlapping JIA and JIA-U is outside of the scope of this study, a recent review of environmental determinants of JIA [31] reported unclear associations between JIA and the risk factors described here. This study provides an important step in identifying shared and distinct environmental influences on JIA and JIA-U, understanding their role in the aetiopathogenesis of these two conditions and designing future studies.

The main strength of this systematic review is its methodology, which was carried out in accordance with PRISMA guidelines [9] with the associated protocol pre-registered on PROSPERO. Since we anticipated limited published data, we sought advice from a database searching specialist to ensure our search syntax was as broad as possible. We hand-searched the reference lists of excluded review articles and used expert opinion to ensure key studies in the field had been captured within our database searches. All stages of screening, data extraction and risk of bias assessment involved a second reviewer. The resource availability for this review necessitated that English language limits were placed on our search strategy, thus it is possible that relevant literature published in another language was not identified. However, the literature included in this review supports the epidemiological data that JIAU is most common in Europe and North America [32]. Since our protocol only allowed for inclusion of full text peer-reviewed articles rather than abstracts/conference proceedings it is possible that relevant ‘grey literature’ was not included. During full text review, 20/67 studies were excluded as data was only available as a conference abstract. Six of these had readily identifiable peer-reviewed manuscripts which were also captured within our database searches. From the data or cohorts presented in the remaining 14 conference abstracts it appears possible that a number of these formed part of a larger dataset which was subsequently published however we cannot be certain. It seems likely that the number of studies that appear only in the ‘grey literature’ (i.e. not published in peer-reviewed journal articles) is likely to be small, however we cannot fully exclude the potential for publication bias. We restricted our study inclusion to those studies examining JIA-U incidence and subsequently limited our data extraction to the same – further work will be required to evaluate the role of environmental risk factors in JIA-U disease activity and/or severity.

## Conclusion

Whilst JIA-U is considered a complex disease influenced by genetics and the environment, this systematic review identified a very limited number of studies investigating environmental risk factors and their association with JIA-U incidence, thus illustrating a paucity of research in this area. The data available suggest an association between JIA-U and vitamin D, and JIA-U and allergy/allergy medications however these results must be interpreted with caution as they represent the output of single studies. Further studies are needed to strengthen the evidence of these associations and explore their contexts.

A co-ordinated and collaborative effort is needed to identify JIA patient cohorts that not only have sufficient ophthalmic follow-up data over a prolonged period to accurately discern JIA-U case status, but that can also analyse putative patient risk factors whilst accounting for potential confounding. International partnerships are likely to be required to generate enough studies to enable meta-analysis of supposed JIA-U risk factors. Undertaking such work to detect JIA patients with modifiable JIA-U risk factors or identify those who may be at particularly increased risk compared to the current accepted JIA-U risk profile would be of considerable benefit to patients and allow more efficient use of health resources for screening.

## Data Availability

All data generated or analysed during this study are included in this published article.

## Abbreviations

ANA: Antinuclear antibody
CINAHL: Cumulative Index of Nursing and Allied Health Literature
JIA: Juvenile idiopathic arthritis
JIA-U: Juvenile idiopathic arthritis associated uveitis
NOS: Newcastle Ottawa Scale
PRISMA: Preferred Reporting Items for Systematic Reviews and Meta-analyses
RF: Rheumatoid factor
Th1: T helper cell type 1
Th2: T helper cell type 2
WOS: Web of Science

## Declarations

Ethics approval and consent to participate.
